# Post‐COVID myocarditis with antiheart antibodies persistence: Clues for autoimmunity?

**DOI:** 10.1002/clc.23979

**Published:** 2023-01-24

**Authors:** Lucas S. Silva, Vinícius O. Boldrini, Ana M. Marques, Clarissa L. Yasuda

**Affiliations:** ^1^ Neuroimaging Laboratory, Department of Neurology University of Campinas Campinas São Paulo Brazil; ^2^ Autoimmune Research Laboratory, Department of Genetics, Microbiology and Immunology, Institute of Biology University of Campinas Campinas São Paulo Brazil; ^3^ Brazilian Institute of Neuroscience and Neurotechnology (BRAINN) University of Campinas Campinas São Paulo Brazil

**Figure 1:**
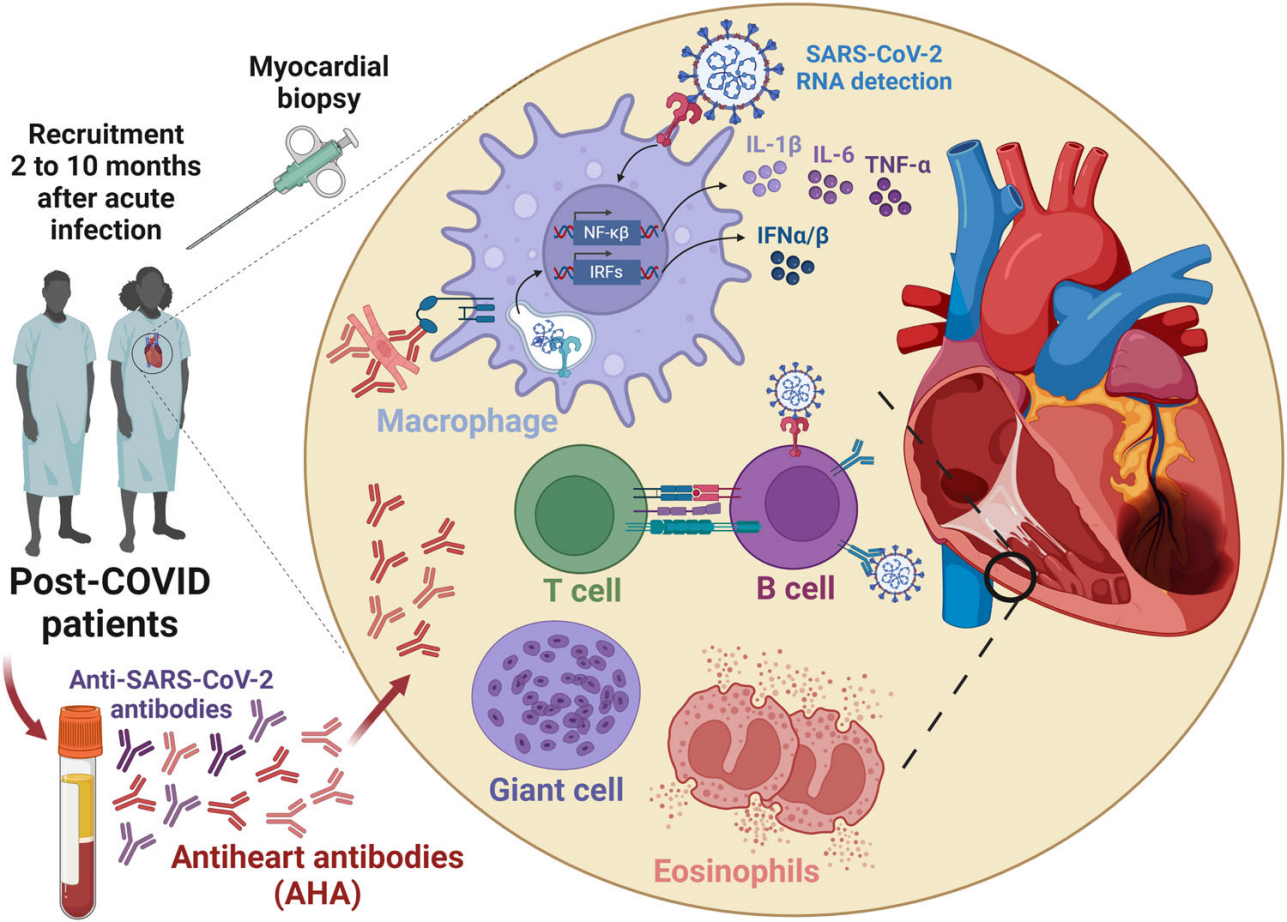
Persistence of antiheart antibodies and other antiviral immune mechanisms eventually involved in post‐COVID myocarditis. SARS‐CoV‐2 structures (e.g.: spike protein, mRNA) might be sensed by surface or endosomal Toll‐like receptors among others pattern‐recognition receptors. Macrophages and other innate immune cells (e.g.: giant cells and eosinophils) that sense SARS‐CoV‐2 may release pro‐inflammatory cytokines (IL‐1β, IL‐6, TNF‐α) and type I interferon (IFN α/β) in the myocardium. B cells may sense SARS‐CoV‐2 through their B‐cell receptor and TLRs. Macrophages and B cells are professional antigen‐presenting cells and can interact with infiltrated T lymphocytes enhancing effector auto‐reactive phenotypes against myocardium autoantigens. Importantly, eosinophils can also release eosinophilic cationic proteins and major basic proteins which are largely described during tissue damage in several conditions. Thus, in association with cellular components, the production and entrance of AHA in the heart may promote myocarditis in the spectrum of post‐COVID manifestations. Created with Biorender.com.

To the Editor,

We read with interest the report by Blagova et al.^1^ and would like to share some ideas and comments regarding the immunological features possibly involved in the post‐COVID manifestations. The authors showed the detection of SARS‐CoV‐2 RNA in 12 of 14 patients exhibiting long‐term post‐COVID‐19 myocarditis (diagnosed 2–18 months after the acute infection). Although these individuals did not present severe respiratory failure or cardiac injury in the acute phase, the biopsies (performed 5.5 months later) revealed viral particles, along with lymph histiocytic elements (with eosinophils and mainly macrophages, T‐lymphocytes, and B cells in the infiltrates). Coronariitis, microvascular thrombosis and parietal thrombi in the right ventricle were also observed. Strikingly, high levels of antiheart (AHA) autoantibodies (auto‐Abs) were detected in 93% of patients. The authors suggested that this could be involved in the physiopathology of post‐COVID‐19 myocarditis (summarized in Figure 1).

Interestingly, Fagyas et al.^2^ recently reported the occurrence of AHA in 68% of patients during the severe clinical course of COVID‐19. In this cohort, the IgM isotype was more prevalent (51%) than IgG (39%). The authors did not find any apparent correlation between COVID‐19 severity and titers of AHA. Besides, increased levels of troponin T were observed in both convalescent and deceased individuals, suggesting myocardial damage during the acute disease.

In another study of post‐COVID sequelae, Su et al.^3^ reported that a percentage of auto‐Abs at the convalescent phase (44%) exhibited a mature profile (class‐switched) at diagnosis (56%) of SARS‐CoV‐2 infection. Of note, about 6% of patients exhibited auto‐Abs before COVID‐19. These findings suggest preclinical antibody production, which raises the question if this production interferes with infectious and postinfectious outcomes, such as triggering autoimmune clinical conditions. Strikingly, a negative correlation between Interferon‐α2 (IFN‐α2) auto‐Abs and anti‐SARS‐CoV‐2 Abs was observed in convalescent individuals after mild infection. One possible explanation is that IFN‐α2 auto‐Abs can dampen IFN‐dependent B cell responses,^4^ and limit the production of neutralizing antibodies against SARS‐CoV‐2. On the other hand, IFN‐α inhibition by auto‐Abs may unbalance self‐tolerance, which in turn may promote auto‐Abs generation.^5^ Finally, there was a positive correlation between multiple inflammatory markers (e.g.: IFN‐γ, C‐Reactive Protein, and IL‐6) detected in the acute stage, and auto‐Abs identified at the convalescent phase.^3^

Altogether, these findings suggest that the emergence of inflammatory markers during the acute phase of COVID‐19 may reduce self‐tolerogenic immune mechanisms implicating in eventual cardiac and other, autoimmune reactions. The early production of auto‐Abs during the acute infection may be related to the physiopathology of distinct manifestations associated with SARS‐CoV‐2 infection.^1^, ^3^ Further, longitudinal immunological evaluation of postinfected individuals may clarify underpinning pathological mechanisms associated with post‐COVID symptoms. A better understanding of the mechanisms involved in postinfection syndrome is essential to developing more specific therapeutic approaches for managing its diverse manifestations.
